# A tree-based explainable AI model for early detection of Covid-19 using physiological data

**DOI:** 10.1186/s12911-024-02576-2

**Published:** 2024-06-24

**Authors:** Manar Abu Talib, Yaman Afadar, Qassim Nasir, Ali Bou Nassif, Haytham Hijazi, Ahmad Hasasneh

**Affiliations:** 1 Department of Computer Engineering, College of Computing and Informatics, University of Sharjah, Sharjah, UAE; 2 Department of Computer Science, College of Computing and Informatics, University of Sharjah, P.O. Box 27272, Sharjah, UAE; 3 Centre for Informatics and Systems of the University of Coimbra (CISUC), University of Coimbra, Coimbra, P-3030-290 Portugal; 4 Intelligent Systems Department, Ahliya University, Bethlehem, P-150-199 Palestine; 5 Department of Natural, Engineering and Technology Sciences, Faculty of Graduate Studies, Arab American University, P.O. Box 240, Ramallah, Palestine

**Keywords:** XAI, Interpretability, Physiological data, Classification, Boosting, Deep neural network

## Abstract

With the outbreak of COVID-19 in 2020, countries worldwide faced significant concerns and challenges. Various studies have emerged utilizing Artificial Intelligence (AI) and Data Science techniques for disease detection. Although COVID-19 cases have declined, there are still cases and deaths around the world. Therefore, early detection of COVID-19 before the onset of symptoms has become crucial in reducing its extensive impact. Fortunately, wearable devices such as smartwatches have proven to be valuable sources of physiological data, including Heart Rate (HR) and sleep quality, enabling the detection of inflammatory diseases. In this study, we utilize an already-existing dataset that includes individual step counts and heart rate data to predict the probability of COVID-19 infection before the onset of symptoms. We train three main model architectures: the Gradient Boosting classifier (GB), CatBoost trees, and TabNet classifier to analyze the physiological data and compare their respective performances. We also add an interpretability layer to our best-performing model, which clarifies prediction results and allows a detailed assessment of effectiveness. Moreover, we created a private dataset by gathering physiological data from Fitbit devices to guarantee reliability and avoid bias.

The identical set of models was then applied to this private dataset using the same pre-trained models, and the results were documented. Using the CatBoost tree-based method, our best-performing model outperformed previous studies with an accuracy rate of 85% on the publicly available dataset. Furthermore, this identical pre-trained CatBoost model produced an accuracy of 81% when applied to the private dataset. You will find the source code in the link: https://github.com/OpenUAE-LAB/Covid-19-detection-using-Wearable-data.git.

## Introduction

Coronaviruses are enveloped by positive, single-stranded giant RNA viruses. This kind of disease initially infects animals and then adapts to infect humans [1]. A recently distinguished coronavirus, SARS-CoV-2, has caused a widespread global epidemic of the respiratory ailment called COVID-19. More than 768,187,000 cases of COVID-19 were recorded until June 2023, with a total death count of 6,945,714 worldwide [2, 3]. The danger of the Covid-19 virus is its ability to spread easily and quickly among humans. Based on the study, the primary reproduction number (R0) estimation has declined to 0.9, compared to its previous value of 1.2 by the end of 2022. However, 0.9 is still a high number, which means there is a good chance that one infected person will infect another [4].

The primary issue that drives our research is identifying COVID-19 infections in the early stages, ideally before the onset of clinical signs. Identifying sick people as soon as possible is critical to protect their health and stop the spread of the disease within communities.

Traditional COVID-19 diagnostic techniques, like antigen testing and Polymerase Chain Reaction (PCR), have shown efficiency in validating infections; however, these techniques failed in preventing the disease from spreading since they can’t detect the disease in its early stages [5]. That was the reason behind the extensive research from researchers worldwide to find strategies to act before symptoms appear. As a result, Different COVID-19 detection methods have been studied, including biometric data, chest X-ray imaging, and blood biomarkers tests for early COVID-19 infection diagnosis and death prediction [6, 7].

Wearable devices and biometric data have recently become essential to solving many medical and physiological problems [8, 9]. Unlike conventional testing, the use of wearable devices offers continuous access to real-time physiological data, which can be used in the early detection of asymptomatic and pre-symptomatic cases of COVID-19 [10–12]. More than 100,000 Fitbit users across the US and Canada have joined a study launched by Fitbit to build an algorithm that detects COVID-19 before symptoms appear. The study produced findings that ensure the credibility and possibility of using physiological data to detect COVID-19 early, helping to lower infection rates [13, 14]. The study finds that an infected person’s resting heart rate consistently increases an average of five to seven days before the onset of the symptoms. In addition, the person’s breathing rate ordinarily crests on day two of symptom onset but generally stays higher than usual for up to three weeks after symptom onset.

According to [14], there is a well-established correlation between Heart rate variability (HRV) and inflammatory conditions, where dramatic reductions in HRV were linked with subsequent rises in C-Reactive Protein (CRP) [15]. On the other hand, many studies show that CRP, a nonspecific inflammatory marker, has an adverse connection with HRV; hence, it can be used as a prognostic indicator to predict the onset of an inflammatory response associated with Covid-19 [16]. Also, according to studies, sleep disturbances are a common symptom for individuals with an acute COVID-19 infection [17]. Where 45% of COVID-19 patients suffer from depression, 47% suffer from anxiety, and 34% suffer from sleep difficulties, according to [18, 19]. Moreover, sleep disturbances such as insomnia have been related to an increased risk of depression and inflammation [20]. Steps count and physical activities, in general, are affected by this disease. Given the clinical traits of COVID-19 [21], infected individuals required to stay in bed cannot carry out regular physical exercise or engage in routine daily tasks. The above studies show helpful links between Covid-19 infection and simple physiological metrics such as HR, step count, and Sleep quality to detect different diseases, including COVID-19.

Looking into the solutions proposed by the previous work to detect Covid-19, we found that most of these solutions lack at least one of the following features, detecting the disease in the early stages at least one or two days before the symptoms appear. And model generalization means testing the model on external data other than the one we trained it on. Those two features will make the model reliable, accurate, and ready to go into the industry.

Building on this foundation, our goal is to extend the applicability of our model beyond a single dataset to increase its reliability by introducing a strategy that uses the concept of transfer leaning to train the AI model on one extensive dataset and then test the same model on another external dataset. This method will increase the model generalization. To identify the disease in its early stages and eliminate its spreading, we decided to use biometric data, considering that it will be the first to change even before the symptom’s onset. Our research critically examines the relationships that exist between COVID-19 infection and the steps count and heart rate (HR) using cross-evaluation techniques to test the model on an external dataset. These correlations offer crucial new information about the early identification of COVID-19.

Furthermore, an advanced and cutting-edge approach known as explainable AI (XAI) was employed to provide transparent and interpretable insights into the decision-making process of AI systems. This encompassed factors such as the quantity and significance of features, techniques for preprocessing data, the influence of diverse models, and the overall effects that these factors exerted on the outputs of the models. Offering explanations and justifications for the outcomes will add trust to the AI model from both the developer’s and end user’s points of view.

Our approach combines Explainable Artificial Intelligence (XAI) technology with physiological data to implement the early detection of COVID-19 and overcome the delay problem caused by other traditional testing techniques. What highlights our work from prior studies is that we introduce the concept of cross-evaluation through transfer learning techniques, enabling the model’s adaptability and consistent performance across multiple datasets. The model is trained using DNN TabNet and custom CatBoost classifiers on a public dataset during this process. To test the model’s reliability and generalization ability, we validated the model using a private dataset different from the training data. Additionally, our solution incorporates XAI technology to improve the system’s transparency and reliability.

The rest of our paper is structured as follows: In the next section, we discuss the related work done in the same field, and later we discuss the data preparation and resources. Section four sets out our methodology, including each model’s overall method and implementation design. Section five discusses our experimental approach and results. Finally, in section six, we conclude our work with a summary and a discussion of future work.

## Related work

Even though the pandemic started more than two years ago, vaccination rates still differ significantly between nations since some countries find it challenging to get enough vaccine doses. Unfortunately, there is currently no confirmed treatment for Covid-19, which emphasizes how crucial it is to continue detecting disease cases and eliminating its spreading.

In this section, we’ll look more closely at the various applications of AI technology for controlling and locating viruses. By utilizing multiple data sources, such as blood test samples, clinical data, physiological data, and medical imaging, scientists and specialists are experimenting with several approaches to detect COVID-19 early.

A detailed overview was done by the authors in [22] that discusses the different data types in COVID-19 detection using deep learning and machine learning. They have included the used data processing and the methodology for each study. They have also discussed the available dataset for this specific task. Many types of inputs were included in the review, such as CT scans, X-rays, cough sounds, MRIs, ultrasound, and clinical markers.

### Image-based solutions

Medical imaging such as X-ray and CT scans have been widely used to detect the coronavirus. Authors in [23–38]and [39] have used x-ray image data to predict and diagnose COVID-19.

Ozdemir et al. [23] proposed using Electrocardiogram (ECG) data to diagnose Covid-19 automatically using deep learning techniques. Hexaxial mapping pictures are produced, and features are extracted using the Gray-Level Co-Occurrence Matrix (GLCM) approach. Their approach achieved 96.20% accuracy and an f-score of 96.30%.

The research conducted by Kaya & Gürsoy [38] used a 3616-sample dataset for detecting covid-19 using X-ray images. They proposed a novel fine-tuning mechanism for COVID-19 infection detection and applied it to a deep transfer learning model. Their best model with the novel fine-tuning approach achieved 97.61% accuracy and reduced more than 81.92% of the total fine-tuning operations.

In [40], the authors used a CNN model with 17 convolution layers that achieved 98.08% accuracy for binary classification and 87.02% for multi-class classification. They used a dataset developed by Cohen JP composed of images shared by researchers with different illnesses. When the study was written, there were 127 x-ray images of patients diagnosed with COVID-19 in the database.

The authors in [25] used a public dataset of chest images divided into three classes: normal, pneumonia-infected, and COVID-19-infected. They used two CNN layers and five max-pooling layers to train their model. They obtained three sets of results: one based on the original dataset, one with a dataset restructured using the Fuzzy technique, and one with a dataset combined using the Stacking technique. COVID-19 data were classified with 100% accuracy, whereas Normal and Pneumonia photos were classified with 99.27% accuracy.

The reference authors [26] used a public access dataset with 5,216 images. They used an Inception Recurrent Residual Neural Network (IRRCNN) and a NABLA-3 network model for classification and segmentation tasks. They used x-ray and CT images to achieve COVID-19 testing accuracy of 84.67% and 98.78%, respectively.

The authors in [41] use a convolution neural network with a strong structure that fits the dataset and the purpose of the model. They combine five publicly available data repositories to source their x-ray datasets, ending with 13,975 CXR images. They obtained 93% accuracy using the COVID-Net model.

Duong et al. [35] used the EfficientNet and MixNet algorithms to detect Covid-19 using X-ray images and CT- scans. They have also used transfer learning to make the approach more efficient. Their algorithm obtained more than 95% accuracy with all the experiments.

Similarly, authors in [36] used an optimization technique called Particle Swarm to automatically fine-tune the deep learning network. Their approach yields an accuracy of 93.9%.

Although X-ray data shows good performance and very accurate results in detecting the lung disorders caused by Corona virus, it still has many drawbacks. The imaging solution is inefficient for long-term tracking and monitoring and impractical for early diagnosis. Moreover, obtaining the required imaging equipment in numerous healthcare facilities can be challenging, and conducting a tomographic study can be pretty costly. Furthermore, the equipment exposes patients to relatively high levels of ionizing radiation. The disinfection process for the CT equipment and the examination room typically takes around 15 min between patients [42].

### Physiological data based solutions

Another AI-based solution has emerged using Blood test samples, gene data, and clinical data, including symptoms, age, etc. Authors in [43] and [44] used blood samples to detect the disease. Their approach gets an F1-score of 78%, and they have also explained their results. Similarly, Yagin et al. [45] have used classical machine learning methods with gene expression data to detect corona virus in individuals. They have used LAME and SHAP to explain their results.

On the other hand, authors in [46, 47], and [48] have used symptom data to detect the virus. Thet used various AI algorithms to obtain the results. IoT technology has also been used to remotely detect COVID-19 cases and engage in early warnings, such as in papers [49, 50], and [51].

The authors in the paper [49] used real-time symptom data from users of wearable device sensors to detect COVID-19 in the early stages. With the help of the Internet of Things and machine learning techniques, the authors could identify potential cases of COVID-19 with over 90% accuracy. The paper proposed a complete detection system using a dataset of 14,251 confirmed COVID-19 cases from the COVID-19 Open Research Dataset (CORD-19) repository, from data collection and processing to develop the machine learning models and AI. They compared the performance of five different ML models.

R. et al. [52] also suggested using IoT technology with data from wearable sensors to predict the occurrence of COVID-19. They collected 238 data samples through wearable sensors and IoT technology. All data was received through a cloud server, with 30 samples labeled as outliers and eliminated, and the remaining 208 samples were used for the research. The data included many biometric parameters to detect the disease, including temperature sensors, heart rate monitors, and respiratory rates. The sensors analyzed the physiological information and transmitted the signals to an ARDUINO controller, which sent the data from the wearable sensor to the cloud server.

In this paper [53], the authors used wearable device data from Stanford University labs to build a model using deep neural network techniques. LSTM algorithm has been used to construct the model. They considered 25 patients with COVID-19 and 11 patients with other illnesses. They preprocess the data using a window of size 8. They achieved an average precision score of 0·91, a recall score of 0·36, and an F-beta score of 0·79.

Miller et al. [54] suggested a way to detect the SARS-CoV-2 virus before the onset of the symptoms using respiratory rate reading. They collected Respiratory rate, resting heart rate (RHR), and heart rate variability (HRV) using the WHOOP strap for 271 individuals. They have trained a gradient-boosting classifier model. Their approach correctly detected 20% of COVID-19-positive individuals before the onset of symptoms and 80% of COVID-19-positive cases by the third day of symptoms.

In a study conducted by M. Gadaleta et al. [55] between March 25, 2020, and April 3, 2021, to show the relationship between heart rate variability and COVID-19, 38,911 individuals enrolled and shared their physiological data. The authors claimed to obtain an AUC of 0.78 when they excluded self-reported symptoms. They used decision trees to prove the concept [55].

Similarly, authors [56] have concluded that prediction using dynamic physiological data may be advantageous for early infection outbreak warning; however, it has some limitations. The authors used a massive dataset collected using Huami devices. The data set includes RHR, activity, and sleep length [56].

Abir et al. [57] also used physiological data from wearable devices, including heart rate and step count, to detect Covid-19. The authors used a Long Short-term Memory Variational Autoencoder (LSTM-VAE)-based anomaly detection framework. Their system predicts 25 out of 25 patients during the infectious period and 80% of the participants during the pre-symptomatic stage [57]. Similarly, authors in [58] and [59] have used body temperature readings to detect Covid-19 and track the disease severity.

Natarajan et al. [60] have also used physiological data such as respiration rate, heart rate, and HRV. Their approach obtained an AUC of 0.77.

In [61], the authors focus on the statistical aspects of the collected data. In the first part of the paper, the authors describe the data they collect using smartwatches from 5262 users. They focus on the participants who wear Fitbit devices.

Many papers use XAI with biosensor data for different applications. In [62], authors use SHAPLY values to elucidate the models’ predictions and explore the impact varying features exert on the models’ outputs. Similarly, Mankodiya et al. [63] use LIME explainable AI models for fall detection applications on wearable devices. Raza et al. [64] also use XAI with federated learning incorporated into the heart health monitoring system.

Previous research primarily focused on using image data for COVID-19 detection, which proved impractical for early-stage diagnosis. Notably, Otoom et al. [49] achieved a good accuracy of 92%, but their results were solely based on symptomatic data, lacking numerical or biosensor information. Consequently, detecting the disease after symptom onset fails to address the crucial issue of controlling its spread. In contrast, our proposed method directly addresses early diagnosis of COVID-19 to mitigate transmission rates. Given the widespread usage of wearable devices, such as smartwatches, in the community, our methodology holds practical implementation potential. On the other hand, as mentioned earlier, many studies have used wearable data to detect COVID-19 disease, proving the data’s significance in promptly detecting the virus [51–57].

Previous studies have covered the detection of covid-19 using various data types and inputs. Many studies have used a combination of chest X-ray and CT scans, deep learning, and CNN neural networks. On the other hand, some research also considered using different types of data such as blood samples, genome, and symptom data that the patients themselves record. Related to our project, some researchers also considered using physiological data from wearable devices and IoT sensors.

However, looking into these studies, researchers face many challenges, especially in the data preparation. When using medical images such as X-rays and CT scans, noise and incompleteness are the leading challenges [22]. When it comes to genome data usage, because the accuracy of existing tests relies on specific sections of the genome, the rapid mutation rate that happened to the genome presents hurdles for diagnostic procedures. Since diagnostic procedures rely on the analysis of genetic changes that frequently alter as the infection develops, mutations raise the possibility of false-negative results [22].

However, our proposed solution is based on the physiological data from accurate wearable devices, with no noise and incompleteness. Moreover, we are using cross evaluation method to double-check our model after the training is done using an external dataset. Our model will be ready for external testing on similar data from different devices in this case. Opposite to the recent studies, our solution proposes detecting COVID-19 early based on the provided physiological data and an explainable AI approach.

Table 1 compares the most closely related studies considering different parameters and characteristics, including evaluating their data, model-building techniques, targeted tasks, and their model interpretation and validation efforts. This table is a valuable resource to highlight our research project’s achievements and how it addresses existing gaps to overcome recent limitations.

**Table 1:** Comparison table of related work: Techniques, Datatype, Tasks, and General information

Ref	Datatype	Task	Technique	Early detection	Explainable AI	Cross-Evaluation	Generalize the model
[44]	Blood test Samples	COVID-19 diagnosis	Classical ML	✖	✓	✖	✖
[31]	X-ray images	COVID-19 diagnosis	CNN + SVM	✖	✖	✖	✖
[45]	gene expressions	identifying COVID-19 gene biomarkers	XGBoost	✖	✓	✖	✖
[47]	Symptoms and basic information	COVID-19 diagnosis	CNN-LSTM	✖	✓	✖	✖
[33]	X-ray images	COVID-19 diagnosis	CNN + SVM	✖	✖	✖	✖
[34]	X-Ray images and CT scan	COVID-19 Multi-Class Classifier	Transfer Learning (DenseNet-121, VGG-16 and ResNet18.)	✖	✖	✖	✖
[38]	X-ray images	COVID-19 diagnosis	Deep Transfer Learning	✖	✖	✖	✖
[23]	Electrocardiogram (ECG) data	COVID-19 diagnosis	Deep Learning	✖	✖	✖	✖
[48]	Temperature and heart rate readings	COVID-19 diagnosis	NA	✓	✖	✖	✖
[43]	blood test samples	Rapid COVID-19 diagnosis	gradient boosting decision tree (GBDT)	✖	✓	✖	✖
[49]	Symptoms dataset	COVID-19 diagnosis	Decision Table algorithms	✓	✖	✖	✖
[53]	Wearable Device data	COVID-19 Early diagnosis	Long Short-Term Memory Networks-based autoencoder (LAAD)	✓	✖	✖	✖
[54]	respiratory rate readings	COVID-19 Early diagnosis	NA	✓	✖	✖	✖
[55]	Wearable Device data	COVID-19 Early diagnosis	Gradient boosting	✓	✓	✖	✖
[57]	Wearable Device data	COVID-19 Early diagnosis	PCovNet, a Long Short-term Memory Variational Autoencoder (LSTM-VAE)	✓	✖	✖	✖
[60]	Wearable Device data	COVID-19 Early diagnosis	Neural Network	✓	✖	✖	✖
Our work	Wearable Device data	COVID-19 Early diagnosis	Tree-Based model with LIME explainable technique	✓	✓	✓	✓

Looking at the table, we noticed a few critical findings that serve our study. Notably, cross-validation was not used in the earlier research to confirm the model’s dependability following the training and testing stages. A primary restriction of this is recognized in works like the ones in references [45, 53], and [47]. Furthermore, while most studies employed Explainable Artificial Intelligence (XAI) to explain their findings using medical images as inputs, the authors in [55] diverged by utilizing a standard feature selection method to demonstrate feature importance.

Another important finding from Table 1 is that most studies relied on blood tests and X-rays taken after the disease was confirmed, indicating that their primary focus was on diagnosing COVID-19 after symptoms appeared, such as [31, 33, 34]. On the other hand, studies [53–55], and [57] have adopted a different strategy and focused on the early diagnosis of SARS-CoV-2, which represents a clear departure in methodology and emphasis from the field’s general tendency.

In contrast to the above-mentioned studies, our research introduces an approach of cross-evaluation, utilizing transfer learning techniques to create a generalized model that can be tested on diverse data sources. The primary goal of this research is to identify the most effective model characterized by the highest scores in evaluation matrices. which aims to create a detection system capable of determining the Covid-19 disease during its initial phases, preventing the uncontrolled transmission of the disease before symptoms manifest.

This study contributes in the following ways:

- We propose an approach utilizing Deep Learning and tree-based machine learning techniques to detect SARS-CoV-2 (COVID-19) at least two days before symptom manifestation.
- Employing transfer learning to generalize the model and ensure its reliability across various biosensor datasets from different sources is a primary limitation in most related work.
- We built a new dataset for COVID-19 detection based on wearable data using Fitbit smartwatches to validate our model.
- We incorporate Explainable AI technologies to elucidate the model’s behavior and highlight important information about the overall data.

## Methodology

### Dataset

In this study, we use two datasets; one is a public database collected by Stanford University labs [65]. The other is a private dataset we collected from Covid-19 patients to validate our model and test its reliability. Many feature extraction techniques have been introduced in this article [66]; however, feature extraction is not an essential step in our methodology since our data is simple tabular data obtained from smart watches and biometric sensors. On the other hand, the procedure for obtaining the readings from the sensors is explained in detail in the dataset section.

#### Public dataset

A study was conducted by the research group from Stanford University Genetics Department [67] on a cohort of participants who completed questionnaires about the symptoms, diagnosis, and severity of respiratory diseases. The study included information from 4642 smartwatch participants, 3325 of whom are Fitbit users. However, only 32 COVID-19-infected Fitbit users had data available throughout their symptoms and diagnosis period [67]. The Stanford dataset, accessed publicly in [68], includes 73 healthy persons, 15 patients with various respiratory disorders, and 32 participants with COVID-19 infection. A specific ID recognizes each of them during the data collection. Only data on COVID-19-positive participants were included in this research project, concentrating on the pre-symptomatic detection of COVID-19 infection [53]. Heart rate and step counts are provided for these participants and information on sleep stage and duration.

Stanford dataset consists of three groups. The first group included participants whose reading was abnormal during the symptom onset. The second group included ten participants for whom researchers from Stanford University detected the disease within 28 days of symptom onset. Group three had six users whose infection periods could not be easily seen using the researcher’s algorithm.

In our work, we used information from 11 participants from the first group to build our dataset since these participants have complete and consistent data. The final dataset consists of 427,866 data readings divided into two classes: 214,022 normal readings and 213,843 abnormal readings. The reading includes heart rate (HR), step count, and sleep data.

#### Private dataset

A team of researchers led by the University of Sharjah in collaboration with the University of Coimbra (Portugal), Arab American University, and Palestine Ahliya University, conducted a longitudinal study. The study involved 28 individuals (with ongoing recruitment efforts) who were asked to wear Fitbit-Versa 2 smartwatches to gather data such as Heart Rate (HR), physical activity (e.g., step count), and sleep patterns. The primary objective was to employ Artificial Intelligence techniques to analyze their physiological and physical activity data for early detection and monitoring of COVID-19 infection, potentially before the onset of symptoms. To ensure ethical compliance, the research group strictly adhered to the University of Sharjah’s ethical committee’s guidelines for non-invasive data collection from human subjects, which were fully met. The initial step involved contacting volunteers through email, personal networks, and social media posts, followed by obtaining their consent, which specified that the collected data would be used exclusively for research purposes and would not be shared or sold to any third party for commercial reasons.

The study hosting platform was created by Dublin City University (AthenaCX, DCU). This private platform enables researchers to quickly create and deploy mobile experience sampling apps (iOS and Android), wearable data collection devices, and integrated consenting. The participants were instructed to download the Athena CX platform, where our application (WeDetect) was made available. They were then instructed to synchronize their Fitbit smartwatches with the Athena CX platform. WeDetect included two types of subjective questionnaires: the first collected demographic information, vaccination history, and disease history (if applicable), while the second was a time-triggered survey with questions related to symptoms and self-assessment. Additionally, the application recorded the wear time of the wearable devices and the response time to the questions. Notably, the study also obtained binary results (positive/negative) of COVID-19 PCR or Antigen rapid tests, along with their respective dates.

**Fig. 1:**
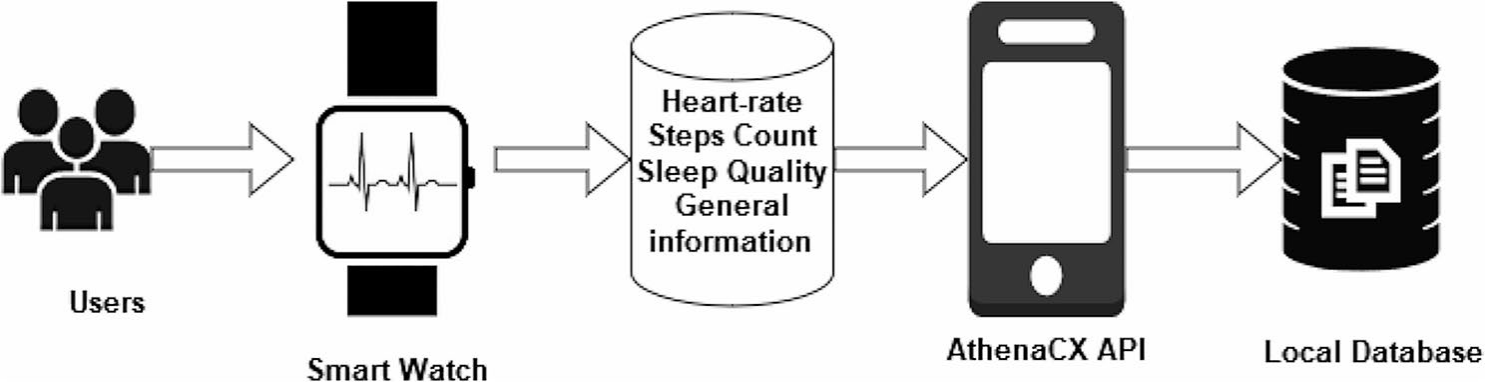
Data acquisition process

Figure 1, the process of data collection, is explained in detail. Firstly, the participants will be asked to download the AthenaCX application, where they find the WeDetect study. Subsequently, their data is transmitted from the Fitbit app through the AthenaCX servers. This data retrieval process occurs on a weekly basis, with the acquired data being stored in our local servers located at UOS.

**Fig. 2:**
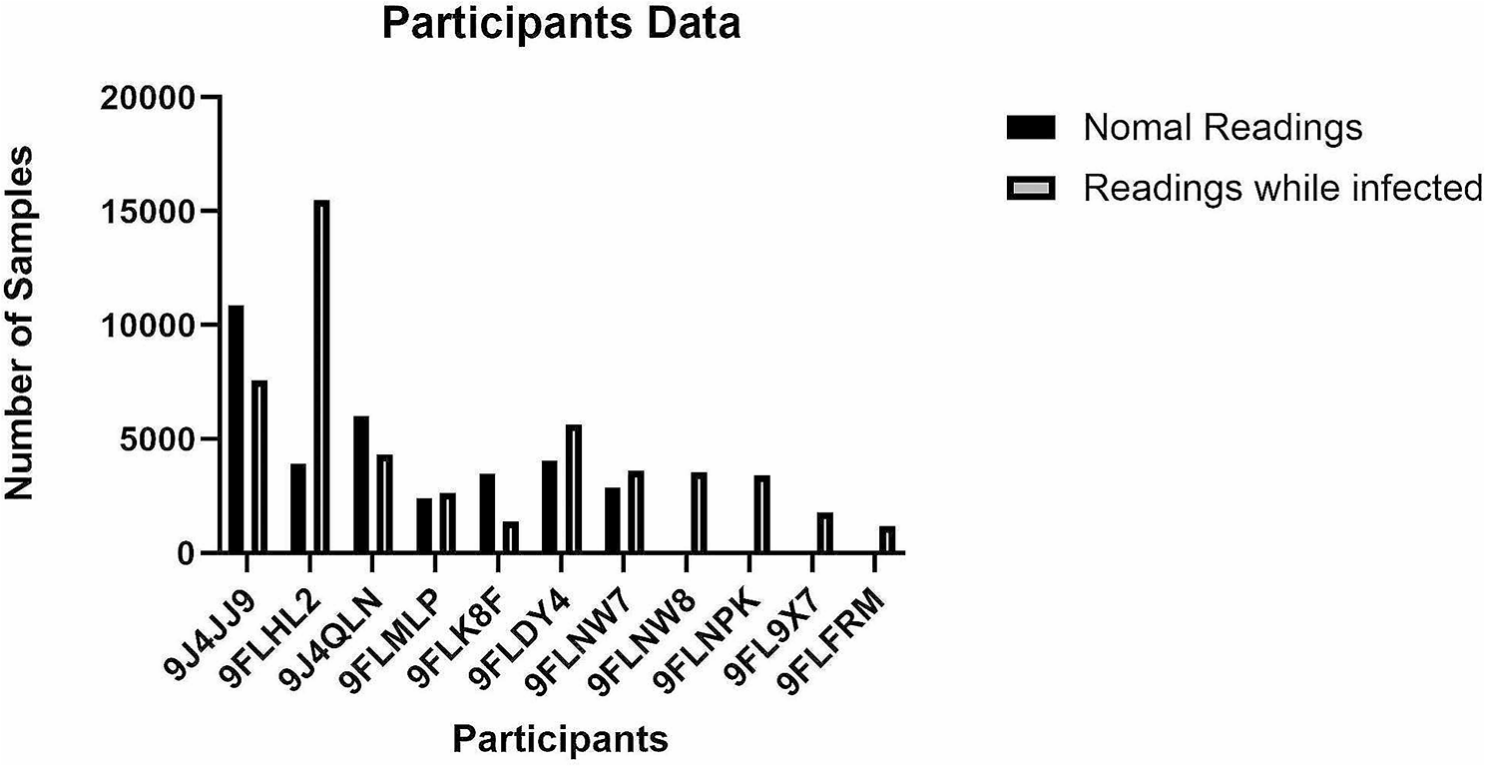
Participants’ reading information for the private dataset

A balanced dataset was constructed by integrating data from 11 participants into a single file. The dataset had 106,847 readings, including heart rate (HR), steps, and sleep data, classified as 50,596 abnormal and 56,251 normal readings. Figure 2 shows How each participant’s data is distributed among the two labels, the healthy and infected patients 9J4JJ9, 9FLHL2, 9J4QLN, 9FLMLP, 9FLK8F, 9FLDY4, 9FLNW7, including both readings before and during they got infected with Covid-19. In contrast, patients 9FLNW8, 9FLNPK, 9FL9 × 7, and 9FLFRM have readings only during the Covid-19 sickness period.

In our work, we used the private dataset to test the model reliability and observe how it would perform in an external dataset other than the training data.

#### Data preprocessing

We applied data processing and feature engineering techniques to both datasets to conduct training and testing, streamlining the learning process and enhancing performance. The data is initially collected in the shape of a timestamp column along with the heart rate and step count features, and in our case, we need to work with the data in the time domain, not the frequency domain; that’s why there was no need to use any type of signal transformations technique such as Fast Fourier Transform (FFT).

**Fig. 3:**
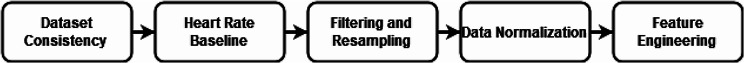
Data processing steps for both datasets

As depicted in Fig. 3, the initial steps involve inspecting data consistency and formatting. Subsequently, we computed the individual heart baseline values for each participant. Following that, we examined our sample pool for missing data and, if identified, as well as removed any outliers. In the final stages, we expanded the feature set through feature engineering.

#### Dataset consistency

The biometric dataset Stanford lab provided is not synchronous regarding the timestamp for the heart rate and the step count. To solve this issue, we ensured that each minute’s heart rate data and the matching step count data shared a common timestamp field.

We also checked for missing or NaN (Not-a-Number) values to preserve the data quality. Although the percentage was not high, we eliminated them with less than 1% missing values from the dataset.

#### Heart rate baseline

The heart rate baseline varies among individuals and is influenced by gender, age, and activity levels.

A couple of steps were applied to find the heart rate baseline for each participant in the study. This baseline reading is the reference point for determining a threshold value that defines a normal heart rate reading.

We gather data by averaging heart rate readings taken during five typical days to compute the heart rate baseline. These readings are collected explicitly during daytime hours, shortly after the participant awakens, from 10:00 am to 10:00 pm. This process allows us to establish a personalized baseline for each participant, crucial for accurate heart rate analysis and anomaly detection [69].

#### Removing outliers by filtering and resampling

Wearable device data is usually produced as one-minute interval readings, including heart rate and step count measures throughout the monitoring session. When we studied this data, we saw abnormalities that lasted for just a minute or two, following which the data resumed a steady trend for the rest of the day. Based on the study [70], a high heart rate with a value of more than 100 beats per minute (bpm) or a low value with less than 30 bpm that lasts for one or two minutes are considered outliers and can be removed, especially when this sudden reading comes in the normal period. According to the study [71], any value that falls outside the 60–100 bpm range is defined as an outlier.

Two distinct methods were used to reduce the effects of these sudden variations:

- **Rolling Averages**: We computed a rolling average for each hour to transform the dataset into an hourly-based format. Because the numbers are averaging over an hour, this method reduces the impact of outliers and sudden variations.
- **Filtering for Normal Labels**: We removed readings from the date when a normal label was determined within the healthy period when both the step count and heart rate values were higher than specific levels. We did not include observations when the heart rate exceeded 100 and the step count was zero. This choice is consistent with a study cited in [70], which states that the range of resting heart rates for all ages is 30 to 100 beats per minute.

#### Data normalization

To enhance our data’s consistency and prepare it for analysis, we normalized it using the StandardScaler method, which was determined to be the most suitable choice after conducting multiple experiments. Normalizing the data yielded substantial enhancements in the model’s performance and led to a notable increase in accuracy scores.

The StandardScaler method is a standardization technique provided by Scikit to learn how to standardize the data, which ensures that the variables have a mean of roughly 0 and a standard deviation of approximately 1. Standardizing the data in general and using StandardScaler enhances the model performance, according to the following study [72].

#### Feature engineering

To enhance the performance of our model, we implemented a final step involving the refinement of the provided datasets. This process involved the expansion of two additional features beyond the existing heart rate and step count measurements. Specifically, we introduced:

- Heart Rate Variability (HRV): Heart rate variability (HRV) is the difference in time between successive heartbeats, and it’s measured in milliseconds [73]. The autonomic nervous system, a basic nervous system component, controls this variation (ANS) [74]. It operates in the background, automatically controlling essential functions like respiration, digestion, blood pressure, and heart rate. It’s also known as RR-Interval. We derive this feature from the HR using the formula: RR-interval = 60/HR.
- Heart rate difference (HRD): This feature is based on an individual’s heart rate baseline. HRD is calculated by taking the absolute difference between the heart rate reading at each minute and the baseline threshold specific to the user. The baseline threshold is computed as the average heart rate over five days when the individual was in a healthy state during the morning period, characterized by zero steps. The HRD value is calculated using Eq. 1.

1 $$HRD = {\rm{ }}\left| {{\rm{ }}H{R_t}-{\rm{ }}H{R_{Baseline}}} \right|$$

Equation 1 Heart Rate difference formula.

By introducing these additional features, we aimed to enrich the dataset and provide our model with more information for improved performance. HRV captures heart rate variations, while HRD reflects deviations from the individual’s heart rate baseline, which are valuable in analyzing and detecting anomalies in heart rate patterns.

### Artificial intelligence models

There are many AI models in this paper that we consider using due to their high performance on our data and due to their algorithm and structure. Since we are dealing with tabular data such as heart rate and step count, we decided to use two strong tree-based models since they outperform classical machine learning and deep learning on normal numerical datasets [75]. On the other hand, we have also used a deep learning algorithm structured for tabular and numerical data, and we decided to include its results.

#### CatBoost classifier

An improved version of XGBoost, categorical boosting, or CatBoost, is used in our research to implement the classification task. CatBoost is a beneficial tree-based model recognized for its precision, quickness, and ease of handling complicated data [76]. Reference [77] emphasizes that some of the best methods for handling tabular data and its complexities are generally boosting tree algorithms. Effective handling of categorical features is one of CatBoost’s main advantages. It processes categorical data directly using a method known as “ordered boosting,” which expedites training and enhances model performance. To accomplish this, we encode the categorical features while maintaining the categories’ inherent ordering.

CatBoost seeks to learn a function F(x) that predicts the target variable y given a training dataset including N samples and M features, where each sample is represented as (x_i, y_i). X_i is a vector of M features, while y_i is the corresponding target variable. Equation 2 describes the math behind the CatBoost algorithms:

2 $$F\left(X\right)= {F}_{0 }\left(X\right)+ \sum _{m=1 }^{M}\sum _{i=1}^{N}{f}_{m}\left({X}_{i}\right)$$

Equation 2 The prediction formula for the CatBoost classifier.

Where:

F(X) is the final predicted output.

F_0_(X) is the baseline prediction.

M is the total number of trees in the structure.

N is the total number of samples in the dataset.

F_m_(X_i_) represents the prediction of the m_th_ tree for the i_th_ training sample.

According to the equation, the initial guess F_0_(x) and the forecasts of each tree f_m_(x_i_) for each training sample are added up to get the overall prediction F(x). The summing process is done for every tree (m) and every training sample (i).

#### TabNet

TabNet, on the other hand, is one of the most robust structures for tabular data, and it uses a deep neural network. TabNet was developed by researchers at Google Cloud AI, and in this study, Pytorch implementation was used [78]. The model employs a sequential attention mechanism to decide which features to consider at each decision point [79]. This model is especially good at handling structured data with many features, making it helpful in predicting prices, classifying items, or making business decisions. It is a suitable compactor for the tree-based structured data models [80].

**Fig. 4:**
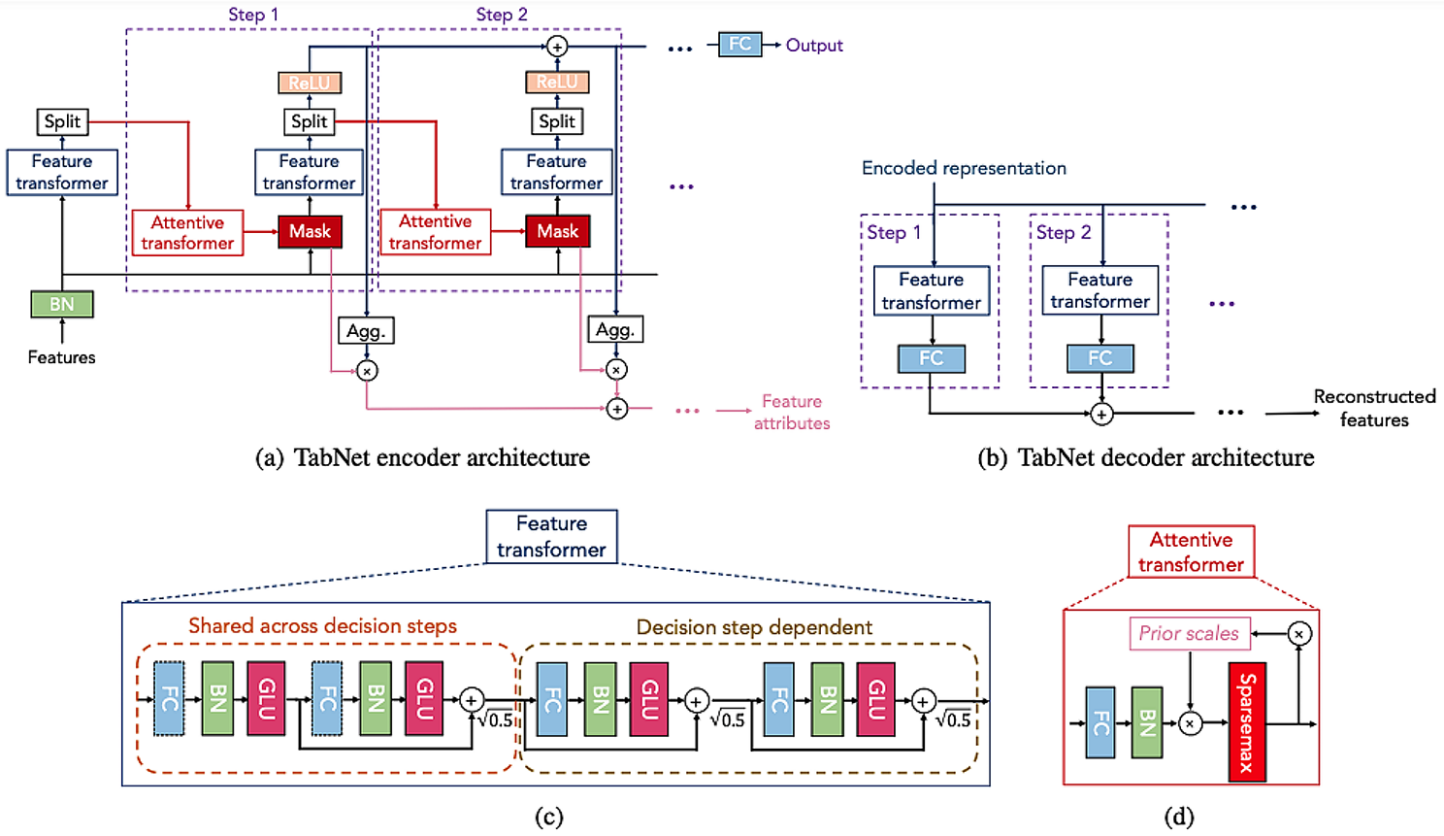
TabNet Architecture from the official TabNet paper [78]

Figure 4 shows the full architecture of the TabNet algorithm, including the encoder and decoder, as well as the feature transformer and the attentive transformer.

TabNet classifier consist of two parts, encoder and decoder. The encoder consists of a feature transformer, an attentive transformer, and feature masking. The feature selection mask offers understandable details about the model’s functioning for every step, and the masks can be combined to produce global feature important attribution [78].

On the other hand, the decoder consists of a feature transformer block at each step [78].

#### Gradient boosting

The last model used in our research is gradient Boosting. The **Gradient Boosting** model is a powerful machine learning technique that builds a robust predictive model by combining the predictions of multiple weak models. It does this by iteratively correcting errors made by the previous models, resulting in an accurate final prediction [81].

The algorithm is based on Eq. 3, which represents the predicting process to minimize the loss L:

3 $${F}_{0}\left(x\right)=argmin \sum _{i=1}^{n}L({y}_{i},\gamma )$$

Equation 3 Gradient Boosting algorithmic formula.

Where argmin means we are searching for the value γ that minimizes ΣL(y_i_,γ), our loss function.

### XAI techniques

Explainable Artificial Intelligence (XAI) aims to make AI systems more transparent and intelligible to humans. Its primary objective is to develop AI models and algorithms that explain how and why they arrived at their conclusions [82]. In this work, we utilized XAI to analyze local points and ascertain the significance of each attribute for each prediction. We used the Local Interpretable Model-Agnostic Explanations (LIME) to characterize our models.

The LIME algorithm, or “Local Interpretable Model-Agnostic Explanations,” is a technique for decomposing complex machine learning models [83]. To do this, it estimates how a model produces predictions for a specific instance (such as a single data point) and provides a concise, intelligible explanation for those predictions. Please find Eq. 4, which represents the LIME formula used to explain the predictions:

4 $$\xi = argmi{n_{g \in G}}{\cal L}\left( {f,g,{\pi _x}} \right) + \Omega \left( g \right)$$

Equation 4 The explanation term for a specific prediction made by a complex machine learning model.

Using the provided equations, LIME tries to find the best interpretable function, g, that minimizes this loss while considering the regularization term Ω(g) [84]. This interpretable function, g, provides a simple and understandable explanation for the complex model’s prediction for a specific data point [84].

### Implementation design

Our experiment is based on cross-evaluation using the transfer learning concept to test the model’s reliability by testing it on a different dataset. Different tuning techniques were applied to initialize the model, which we will discuss in detail later in this section.

All experiments were performed with a 70%, 15%, and 15% ratio of training, testing, and validation for the Stanford dataset. In contrast, we used the full private dataset to test the models after fully training them and get the checkpoints. An early stopping callback was implemented using the validation data with a patience parameter of 10 epochs. So, the training will automatically terminate when the validation loss does not improve after ten epochs. K- fold Cross-validation was used with k equal to ten, where K is the number of groups in which the data will be split [85]. Fine-tuning was applied to the parameters for each algorithm, which is explained in detail in the next paragraph.

**Fig. 5:**
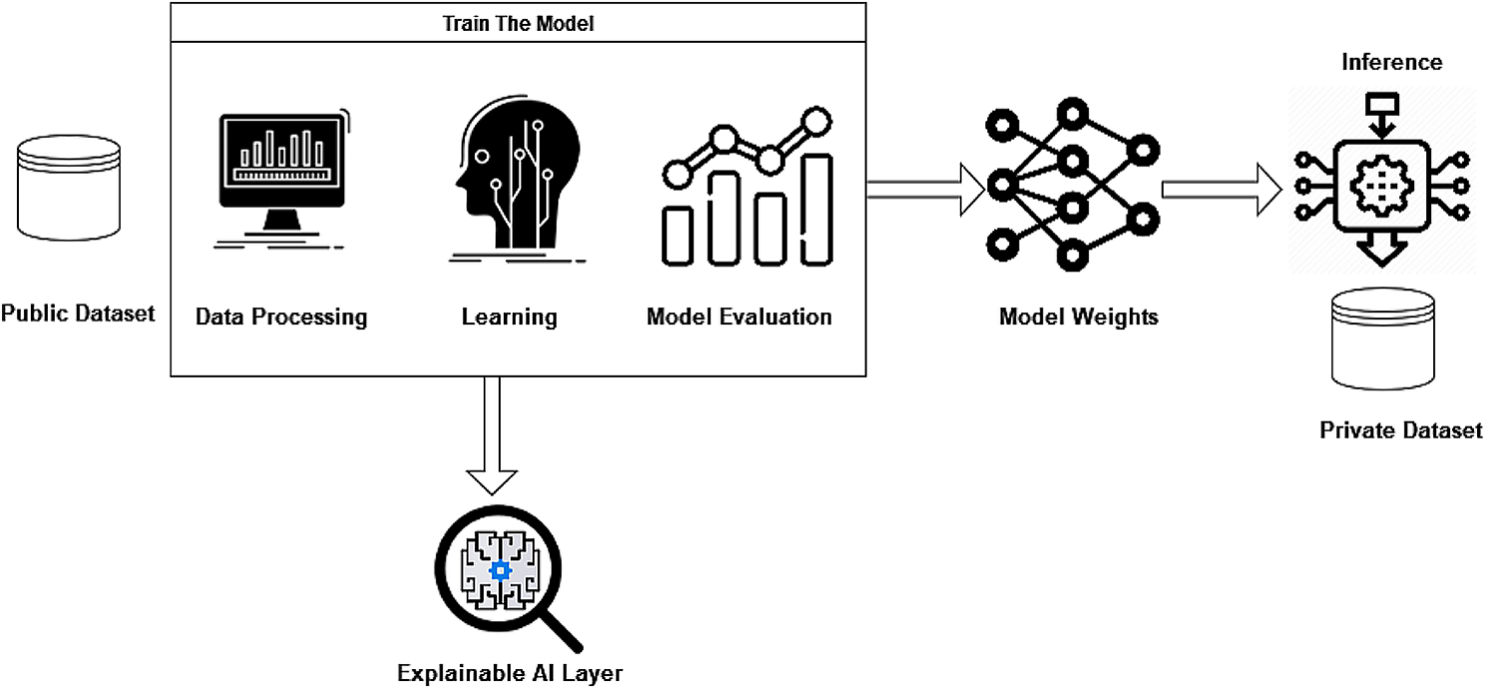
Overall methodology diagram

Figure 5 explains the methodology followed in this research paper. As a first step, model training, and initial testing were done using the public dataset from Stanford labs. An explainable AI technique was used to analyze the model predictions and highlight the important information from the input data. The checkpoints were saved and reused for testing purposes on the private dataset.

**Fig. 6:**
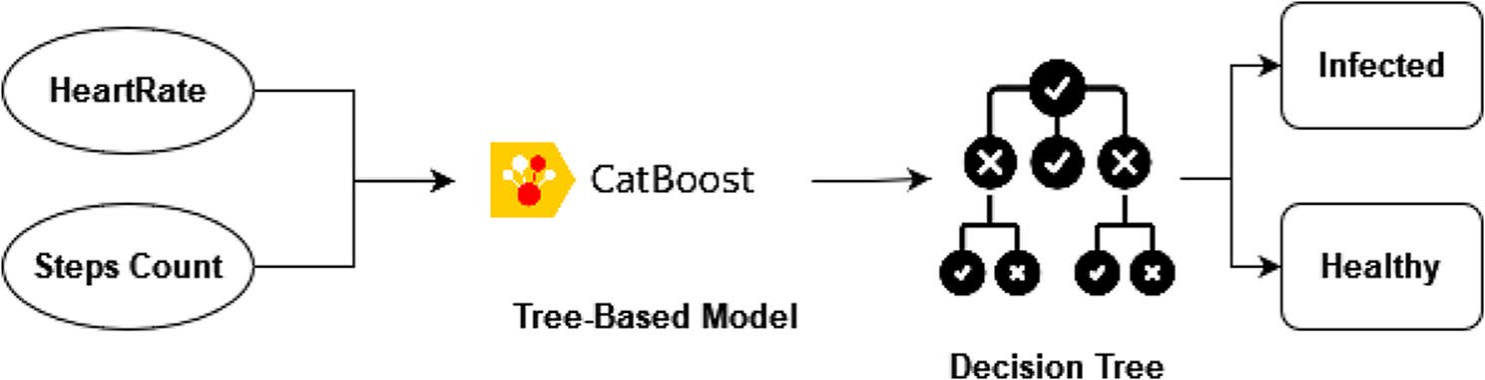
Tree-based model methodology

Since our main focus is to build a strong tree-based model to predict the event of Covid-19 from the basic physiological data, the CatBoost model is used without any time-series model. Find below Fig. 6, which explains our methodology focus:

As shown in the above figure, the model will be capable of predicting the event of Covid-19 using only the physiological parameters without the need for the timestamp feature, which makes the model practical and reliable. Since in a real-life scenario, only physiological data will be provided without looking at the time flow.

### Parameters tuning

Before using the models, we try to fine-tune the parameters for each model to optimize model performance by finding the right balance between underfitting and overfitting. It systematically adjusts model parameters, learning rates, and architecture to enhance generalization to unseen data.

The CatBoost produced good results, and we found it an excellent approach to dealing with large-scale tabular data. In the successful experiment, we set the learning rate to 0.3 and the depth to 8. We choose the value after many initial trials and observe the result in each run. We iterated 350 times to get the best result. We set the evaluation matrix to AUC and obtained the confusion matrix parameters [86, 87].

For the TabNet implementation, we set the learning rate to 2e-2 with the Adam optimizer. We set the maximum epochs to 80 while considering the early stop function with patience equal to 15.

The grid search optimizer is applied to dynamically find the optimal parameters to train the classifier for the Gradient Boosting implementation.

Table 2 presents the parameters that require fine-tuning, each associated with a specific range of values. These values from Table 2 are then utilized as inputs for the grid search optimizer, facilitating the discovery of the optimal combination of values that results in the highest model performance.

Among the parameter choices outlined in Table 2, the following values were determined as optimal for boosting: ‘n_estimators’: 200, ‘learning rate’: 0.1, ‘max depth’: 8, and ‘min_samples_split’: 1000.

**Table 2:** Parameters range for the GC optimizer

Parameter	Values range
Number of estimators	[50, 100, 200, 300, 400]
Learning Rate	[1.0, 0.5, 0.1, 0.01]
Max depth	[6, 8]
Minimum sample split	[500, 1000]

### Models testing and validation

We employed a transfer learning approach to validate our research and assess its reliability. This process involved saving the weights of the four models after successfully training using the Stanford dataset. Subsequently, these saved model checkpoints and weights were utilized in a separate experiment involving a private dataset collected by UOS University. The fundamental idea behind transfer learning is to leverage the knowledge gained during the training on the Stanford data and apply it to predict results for a completely new dataset.

The workflow encompassed several key steps:

To confirm the reliability of our findings, we employed a transfer learning approach. After the four models were successfully trained on the Stanford dataset, their weights were saved as an h5 file. After that, these model checkpoints and weights were used in a separate experiment using a unique dataset obtained by UOS University. The fundamental idea behind transfer learning is using the skills learned during training on the Stanford dataset to predict outcomes for a completely new dataset.

The procedure comprised several important steps:

#### Training and model saving

We kept the model checkpoints and their associated weights after finishing the models’ first training on the Stanford dataset. The information learned throughout the training process was contained in these preserved models.

#### Preparing the private dataset

We preprocessed the private dataset that UOS collected to guarantee compatibility and promote a smooth transfer of information. The data had to be shaped to fit the Stanford dataset’s feature pool and format.

#### Loading pre-trained models

The pre-trained models were put into the experimental setup with their saved checkpoints and weights. With the new dataset, these models were prepared for prediction tasks.

#### Testing with new dataset

The loaded pre-trained models were then employed to predict outcomes for the UOS University’s private dataset. This testing process aimed to evaluate how well the knowledge gained from the Stanford dataset could be transferred to a distinct dataset. The results of this testing phase were crucial in assessing our models’ reliability and generalization capabilities to detect COVID-19 based on physiological data.

Next, the pre-trained models were loaded and used to forecast results for the private dataset owned by UOS University. This testing procedure aimed to determine how successfully the knowledge achieved by the Stanford dataset might be applied to a different dataset. In evaluating the dependability and generalizability of our models to identify COVID-19 based on physiological data, the testing phase findings were critical.

In conclusion, transfer learning enabled us to effectively use the knowledge we had gained from training on one dataset to generate predictions on an entirely different dataset, demonstrating the resilience and adaptability of our models in the context of COVID-19 identification.

## Experiment setup and results

Our approach focuses on applying deep learning and boosting tree models, specifically GB and CatBoost from boosting trees and TabNet from deep learning. The Stanford dataset, which provides a large amount of patient data, was used to train and evaluate these models. We used transfer learning by utilizing computed weights for predictions to guarantee the models’ applicability to a wider range of physiological data outside the training set.

For this research, we conducted our experiments on a computing system and software environment equipped with the following specifications:

- GPU: NVIDIA GeForce RTX 2070 Super with Max-Q Design.
- RAM: 64 GB DDR4.
- Processor: Intel(R) Xeon(R) W-10,855 M CPU @ 2.80 GHz 2.81 GHz.
- Operating System: Windows 10.
- Programming Language: Python 3.8.
- Machine Learning Frameworks: [TensorFlow, Scikit-learn]

### Models performance

Table 3 presents the results of the evaluation metrics—accuracy, precision, recall, and F-score—using three different models. The table is separated into two sections: the first part displays the findings from the public dataset used for training and testing, and the second part displays the results from the private dataset’s validation procedure.

**Table 3:** Result for the public and private datasets using the four algorithms

Dataset	Models	Accuracy	Precision	Recall	F-score
Public Dataset	TabNet	0.78	0.62	0.91	0.73
	CatBoost	0.85	0.83	0.81	0.81
	Gradient Boosting	0.81	0.83	0.81	0.81
Private Dataset	TabNet	0.47	0.99	0.54	0.69
	CatBoost	0.81	0.79	0.77	0.78
	Gradient Boosting	0.48	0.60	0.51	0.55

Most models did well when testing with the Stanford dataset. The main challenge was obtaining close evaluation scores when testing the model on the private dataset, as it was not part of the training process.

## Discussion

The applied algorithm has merits and demerits concerning performance, complexity, and model reliability. High detection accuracy and early disease detection are two important advantages of the provided approach. We also introduced a clear explanation for our models, ensuring their reliability and model interpretability. The use of wearable device data makes the approach user-friendly, as obtaining this data type is relatively easy and inexpensive compared to other data types, such as X-ray images, CT scans, genetic data, etc.

On the other hand, the model’s performance might be impacted by concerns about the security of data from wearable devices. Moreover, the lack of physiological matrices such as SPO2, breath rate, and skin temperature makes the model less unfailing. Having access to this data will increase the performance.

**Fig. 7:**
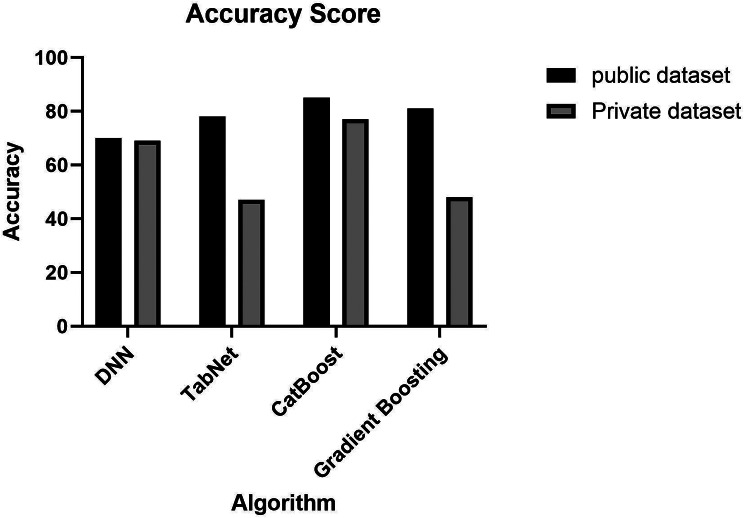
Comparison between accuracies for the two datasets using the four models

In Fig. 7, we can see a big difference between the performance of the public and private datasets using the TabNet algorithm, with an accuracy of 78% and 47%, respectively. Same with Gradient Boosting, with around a 33% difference in accuracy between the private and public datasets. On the other hand, the Catboost algorithm obtained relatively excellent results on both datasets with less than a 7% difference between both accuracies. This makes it the most reliable model as it reaches the experiment’s primary objective.

To prove the above discussion, T-tests were applied to compare the performance of each model among the two datasets and ensure the significance of the CatBoost model among the three suggested models since it performs well on both datasets. T-tests are a statistical analysis used to compare the means of two groups and ascertain whether any observed differences are statistically significant or simply due to chance.

Table 4 provides the results of T-tests with a 0.05 significance level conducted to determine if there is a statistically significant difference in the performance of the models between the two experiments.

**Table 4:** T-test results for the models to prove the difference in performance between the two experiments

Model	*p*-value	Result
TabNet	0.640	Fail to reject the null hypothesis
CatBoost	0.001	Reject null hypothesis
Gradient Boosting	0.071	Fail to reject the null hypothesis

**Fig. 8:**
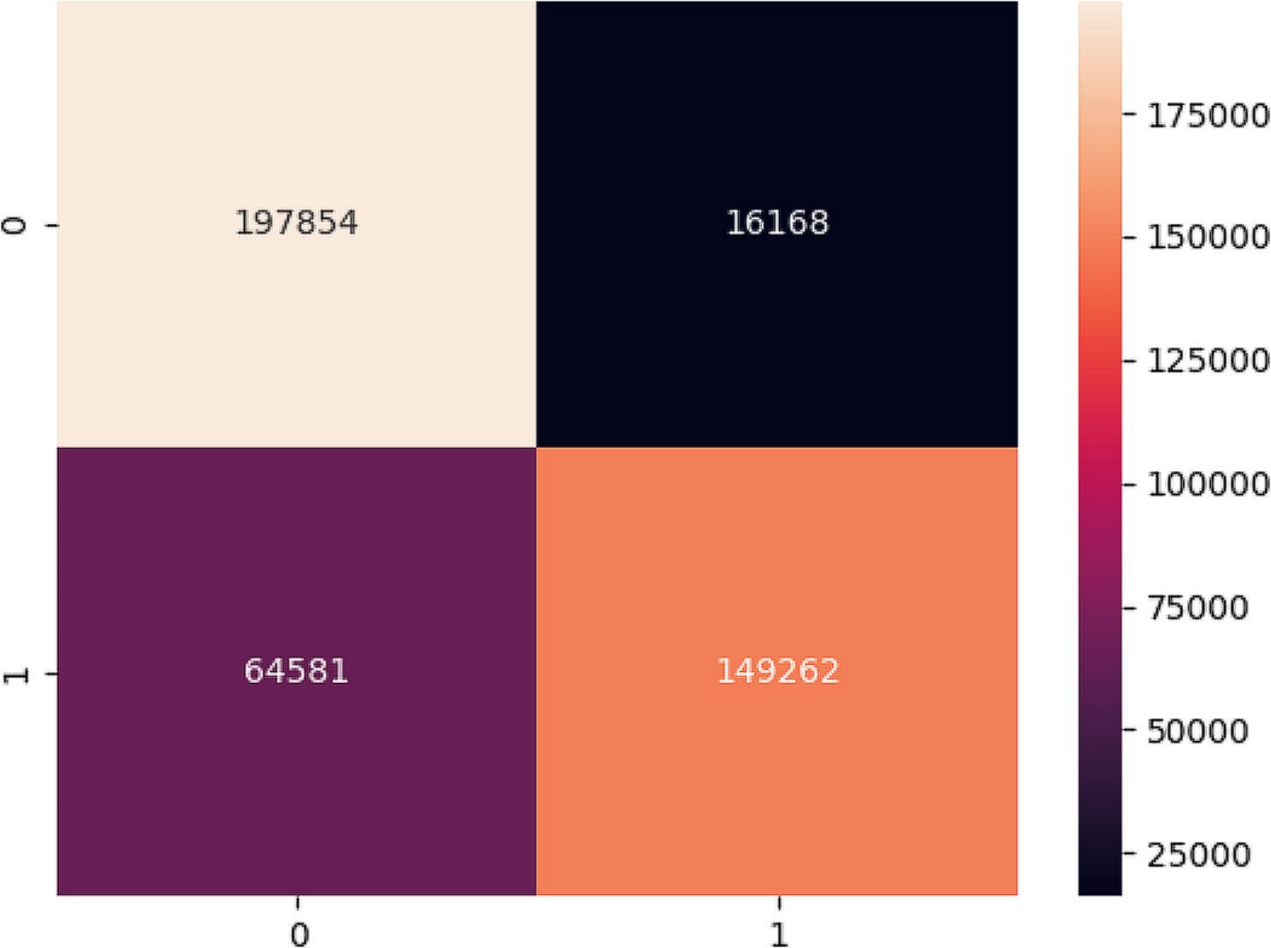
Confusion matrix for the CatBoost model using the public dataset

As shown in the table, the test failed to reject the null hypothesis for both models, TabNet and Gradient Boosting; this proves that there is a difference in performance between applying the results on the public and the private data; however, the test rejected the null hypothesis for the CatBoost model. Assuming that the null hypothesis is that there is a difference in performance between the public dataset and the private dataset using the same model,

The confusion matrix below in Fig. 8 shows the distribution of the false and true predictions regarding one reading instance using the Stanford Laba dataset. We can see that most of the wrongly predicted data points fall under the false negative, which means the model predicts 19% of the instances as healthy, given that these instances were infected.

**Fig. 9:**
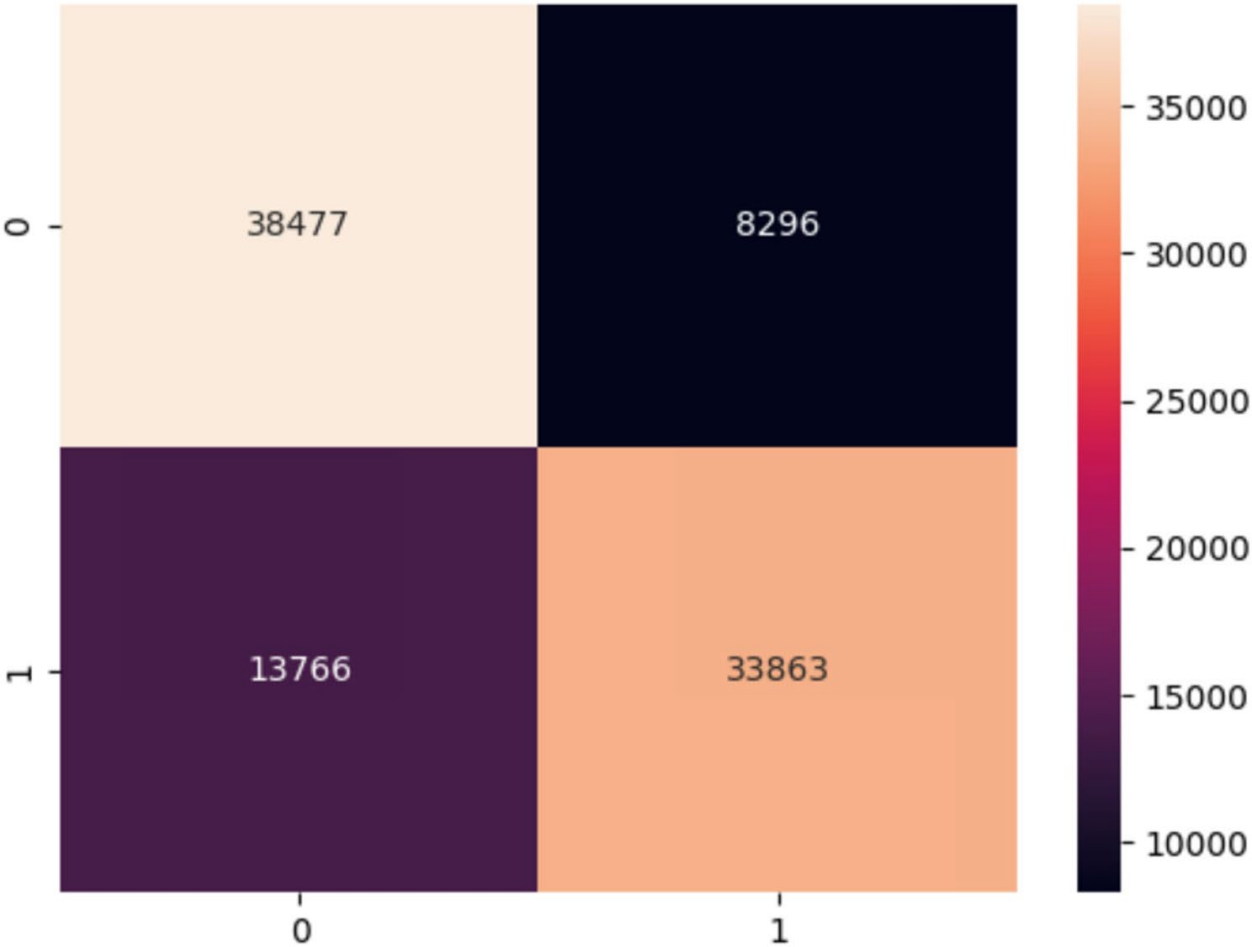
Confusion matrix for the CatBoost model using the private dataset

Figure 9 represents the confusion matrix that shows how the model performed after loading it to test it on an external dataset that was not trained on.

The confusion matrix shows that the pre-trained model performs well after testing it with private data.

**Fig. 10:**
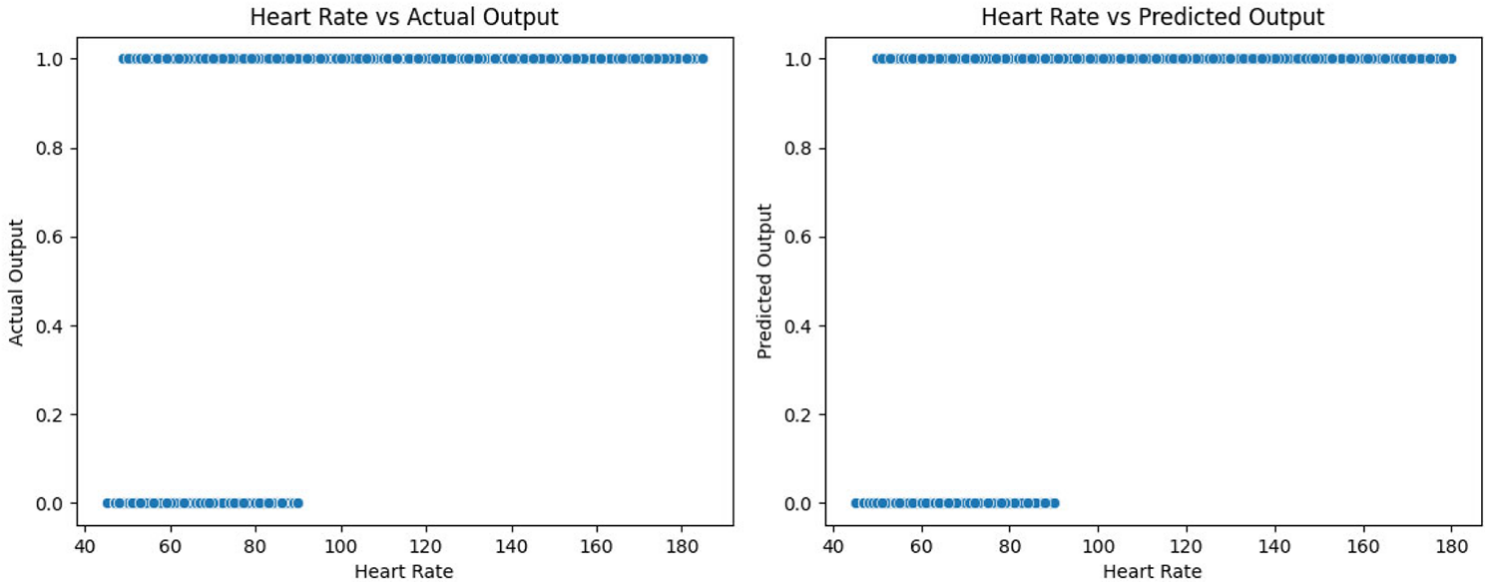
Heart rate reading distribution among labels

Figure 10 shows how the heart rate readings were distributed among the two classes. At first, we can see from the plots that the actual and predicted output have the same heart distribution among classes, which assures the model’s reliability. Looking into the second plot, we can observe that the model put a threshold on the heart rate reading.

In Table 5, we present a comparative analysis of our research alongside existing methods for COVID-19 detection. This table summarizes key findings from related papers. It specifies the data used for each article, their technique to build their model, the overall accuracy and F-score, and the XAI method used to explain the model outcome.

**Table 5:** Comparative analysis of our results against existing methods

Paper	Data type	Technique	Accuracy	F-score	Early Detection	XAI method
[31]	X-ray image	CNN	0.99	0.98	✖	✖
[33]	X-ray image	CNN	0.99	NA	✖	✖
[38]	X-ray image	MobileNet-based CNN	0.97	0.97	✖	✖
[43]	images	Ensemble learning	0.80	NA	✖	LIME
[35]	CT images	Deep Neural Network	0.95	0.938	✖	✖
[36]	images	PSO + CNN	0.93	0.93	✖	Grad-CAM
[47]	Symptoms data	CNN-LSTM	0.85	0.85	✖	LIME
[49]	Symptoms data	SVM	0.92	0.93	✖	NA
[53]	Physiological Data	LSTM	NA	0.79	✖	NA
[55]	Physiological Data	Gradient Boosting	0.78	NA	✖	SHAP
[57]	Physiological Data	LSTM	NA	0.89	✖	NA
[60]	Physiological Data	Logistic Regression Model	0.77	NA	✓	✖
Our	Physiological Data	CatBoost	0.85	0.81	✓	LIME

Compared to the above-summarized research papers, our study focuses on physiological data. It utilizes the CatBoost technique to detect COVID-19 early, achieving an accuracy of 85% and an F-score of 0.81. What sets our research apart is the application of transfer learning for model generalization and the adoption of the LIME XAI method to explain our model’s outcomes. This combination of techniques and methodologies demonstrates our unique approach to COVID-19 detection, allowing for robust results and Interpretability in our model’s predictions.

The researchers in [31, 33, 35, 36, 38, 43, 47], and [49] utilize both images and symptoms data, indicating their emphasis on diagnosing COVID-19 after the onset of symptoms. This is evident from their reliance on symptom data and X-ray images obtained post-confirmation of the disease. This finding explains their high results. However, detecting the disease in its early stages is more crucial than diagnosing it.

Moreover, our method stands out as the top-performing approach when compared to other studies that utilized physiological data to early detect Covid-19;. However, authors in [57] obtained an f-score of 0.81, on the other hand, the *recall on their work is 0.3, signifying a substantial number of false negatives* – instances where the model failed to correctly identify actual positive cases. In contrast, our method achieved a notably higher recall of 0.81, indicating that we successfully captured a larger proportion of the actual positive cases, resulting in fewer false negatives.

### Explainable AI discussion

**Fig. 11:**
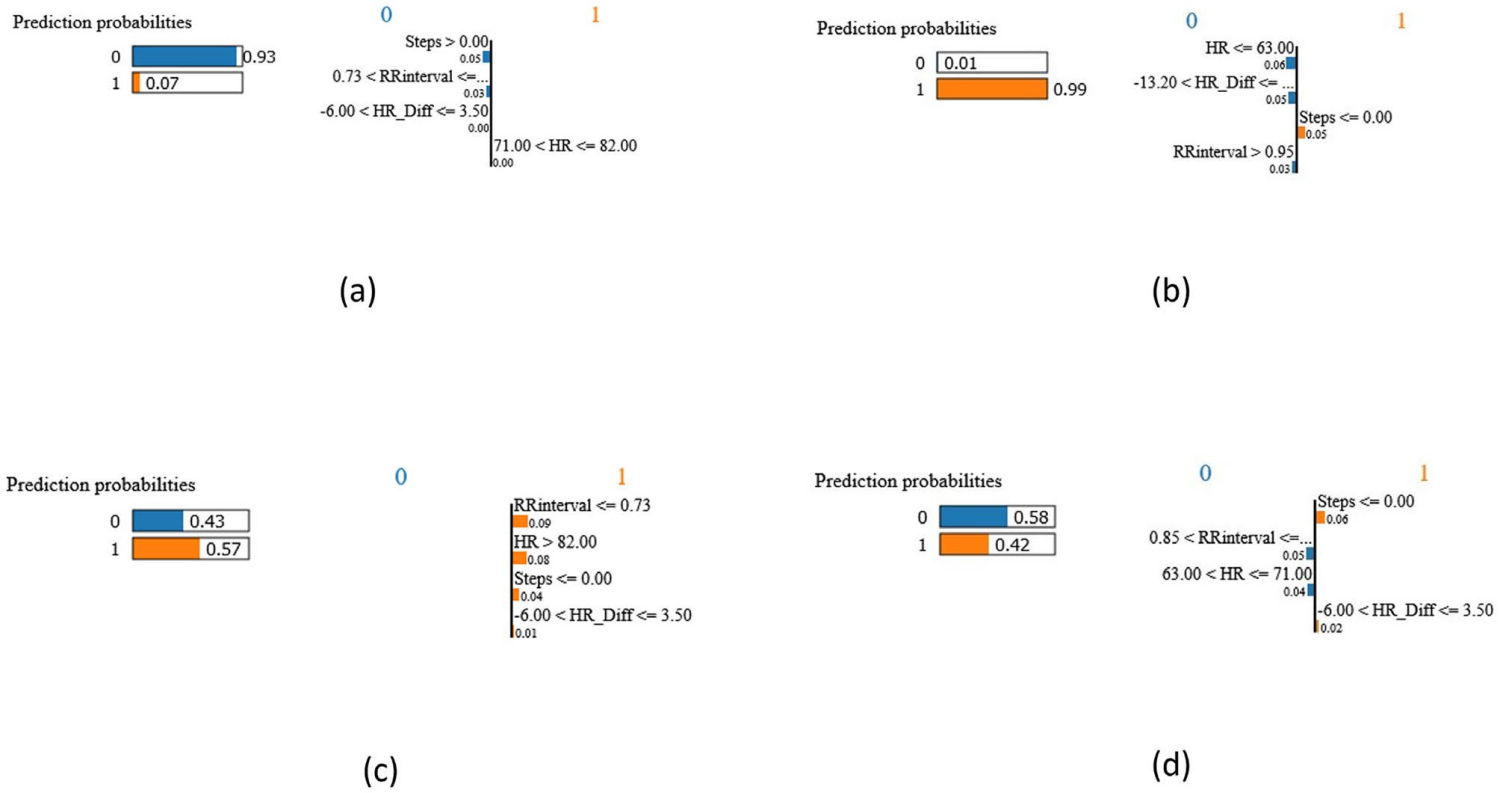
LIME output for four prediction cases, True positive, Tru negative, False positive, and False negative represented as **a**, **b**, **c**, **d**, respectively

Utilizing the LIME method, we gained insights into the model’s behavior at a local level, allowing us to understand the specific features contributing to both correct and incorrect predictions. Figure 11 presents four distinct test cases representing true-positives, true-negatives, false-positives, and false-negatives, respectively.

Subfigure (a) in Fig. 9 denotes a true positive, meaning that the model correctly predicted and identified an individual as having COVID-19. The strong confidence of the model is shown in the high probability of 0.99 assigned to this prediction. Decision rules considered steps, heart rate (HR), and heart rate variability (HRV); the HR was 63.00, the steps were larger than 0.00, and the HRV was less than or equal to 0.73. Together, these guidelines allowed for accurately identifying a COVID-19 case using the individual’s physiological data. Similarly, with a high probability of 0.93, the model accurately predicted the lack of COVID-19 in the subject, as shown in subfigure (b).

In the False negative (d), the model incorrectly predicted that a person did not have COVID-19 when they did. The predicted probability was moderate at 0.57. The decision rules involved steps less than or equal to 0.00, HR greater than 82.00, and HRV less than or equal to 0.73. Despite the incorrect prediction, the probability was not exceptionally high, similar to the false positive (c) scenario.

To conclude this experiment, we can say that the high prediction probabilities in True Positives and True Negatives indicate robust and accurate predictions. However, the model’s performance is not flawless, as the False Positives and False Negatives demonstrated.

These cases highlight instances where the model made incorrect predictions, possibly leading to unnecessary concerns (False Positives) or missed diagnoses (False Negatives), which is happening due to the absence of SPO2 and oxygen saturation features, which we acknowledge as a limitation in our study, as discussed in the following section.

The lower probabilities in these cases reflect the model’s reduced confidence. Therefore, while the model excels in many instances, there is room for improvement, especially in reducing the False Positives and False Negatives rate to enhance its overall accuracy and reliability.

## Conclusion and future work

The primary goal of this research is to identify the most effective model characterized by the highest scores in evaluation matrices. This aims to create a detection system capable of determining the Covid-19 disease during its initial phases, preventing the uncontrolled transmission of the disease before symptoms manifest.

Transfer learning gives our model extra value since it could be tested and validated on any external dataset coming from wearable devices, making it a powerful solution for research and experimentation. AI models, including Deep Neural Network and Tree-Based models, were implemented to achieve the project target, and results were recorded and compared. Our classifier is based on deep neural networks designed through experimentation.

The comparison concludes that Tree-based models outperform Deep learning models, with accuracies of 0.78, 0.85, and 0.81 using TabNet, CatBoost, and Gradient Boosting, respectively. Using XAI, we were able to highlight the critical features of each label and analyze the individual predictions to give better insight into the model. Our methodology was verified on a private database that we collected using the same smartwatch devices to avoid bias, and it resulted in a comparable performance when using the CatBoost algorithm. Moreover, the XAI technique was also introduced to ensure model reliability and explain the results.

Oxygen saturation and SPO2 levels were not accessible to developers from the Fitbit application; we consider this a limitation since those two features are significant to detect COVID-19, and adding them will significantly improve the work.

More related features could be added to the feature set, such as oxygen saturation, breath rate, and skin temperature, to increase the model’s reliability. The approach could be implemented and studied in various disease detection since our features are very general and could be used for any disease diagnosis. Moreover, time series techniques and deep learning techniques will be considered for further exploration along with the traditional machine learning techniques. Furthermore, new methods will be introduced to enhance accuracy and performance.

Conducting classical statistical methods to explain the models instead of using XAI techniques will also be an excellent point to have as a baseline.

## Data Availability

Due to privacy and ethical concerns, the private dataset cannot be made available to the public. Please contact the corresponding author for further information about the data. Please find the link to the Source Code: https://github.com/OpenUAE-LAB/Covid-19-detection-using-Wearable-data.git Find the link to the public dataset from Stanford Labs: https://storage.googleapis.com/gbsc-gcp-project-ipop_public/COVID-19/COVID-19-Wearables.zip
